# Female influencers: Analyzing the social media representation of female subjectivity in Italy

**DOI:** 10.3389/fsoc.2022.1024043

**Published:** 2022-09-21

**Authors:** Geraldina Roberti

**Affiliations:** Department of Human Sciences, University of L'Aquila, L'Aquila, Italy

**Keywords:** gender, social media, patterns of femininity, female influencers, feminism, followers, Italy

## Abstract

The paper addresses the representation of female subjectivity on social media, highlighting how such women's images have a significant impact on the gendered narratives and discourses that populate the public sphere. The article analyzes specifically the pattern of femininity represented by female influencers and the bond they are able to establish with their followers. Starting from the notion of post-feminism sensitivity, it highlights how these celebrities embody its ideals in terms of self-realization, independence and empowerment. Finally, the figures of two of the most popular social media influencers in Italy, Chiara Ferragni and Benedetta Rossi, are analyzed, highlighting their ability to represent a successful model of female digital entrepreneurship. The analysis aims to point out how these media personae provide an ambivalent model of female subjectivity, because on one hand they emphasize women's ability to assert themselves as ambitious, professionally autonomous and free-choosing subjects (as postfeminist culture prescribes), but on the other hand they exploit the spur to female self-fulfillment for marketing aims, turning it into a self-branding tool.

## Introduction

In a quickly transforming social, economic and cultural context, the impact of the epidemic caused by the spread of the Covid-19 virus has contributed to deeply modifying social dynamics, bringing about important changes both in the process of defining subjects' identities and in the ways of interacting and creating meaningful social relationships. In some ways, lockdown period has made even more evident that overlap between offline and online space that characterizes contemporary societies and that makes the boundaries between the two dimensions increasingly blurred. Sociologists and communication scholars have been long pointing out how individuals are moving with increasing confidence in such an ecosystem, experimenting with interaction models that seamlessly combine the activities carried out in the two spheres. Driven by a logic that privileges the continuity of the communicative experience, rather than the separation of identities, networks or communication patterns (Marinelli, 2011), social actors adopt sociability practices that hold together the online and face-to-face dimensions, building relational networks based on choice and emotional similarity.

When, a few years ago, Boyd (2008) drew attention to the progressive “context collapse” linked to the spread of social media, she pointed out how this process affected the dynamics of self-presentation and impression management performed by social actors^1^, who interact through networks that bring together commonly distinct audiences (see also Marwick and Boyd, 2011). The rise of such mechanisms has prompted researchers to investigate with increasing attention the interaction processes enacted online, so as to better understand not only the social bond-building dynamics, but also the role played by digital platforms in the production of new collective imaginaries.

It is clear how the analysis of these phenomena is also connected to the reflections arising within the field of gender studies, since media continue to represent a kind of repository of social models and depictions with which women, in particular, have to deal on a daily basis (Buonanno, 2014). If, as Gill (2007a) wrote, in public discourse there is now a strong focus on the contradictory nature of gender constructions, at the same time it appears necessary to question how the interrelations between media and gender produce new forms of subjectivity, imaginaries and models of action. From this point of view, the commitment to the shaping of different female identities must come to terms with the images of women proposed by the communication system, leading scholars into the field of gender and feminist media studies to investigate the positive elements, as well as the risks, of this interrelation^2^. As Mendes and Carter (2008, p. 1701) write, “[...] feminist communication scholarship always has at its core a goal of examining how gender relations are represented, or the ways in which audiences make sense of them, or how media practitioners contribute to perpetuating sexual inequalities”. This entails reflecting on the function of media in actively contributing to the collective imaginary and social roles production, and thus understanding how socially constructed gender roles can affect one's individual life chances and sense of self-worth.

The fast spread of digital media and—specifically—of social media has made this scenario even more complex, since it has multiplied the possible role models, but also the surveillance mechanisms with which, especially women, have to deal. In a recently published article, Gill (2021), reporting on the results of a survey conducted on a sample of over 200 young people between the ages of 18 and 30, pointed out how the interviewees reported the difficulty of always having to live up to the models normatively conveyed by social media^3^, also emphasizing their prescriptiveness with respect to social roles. Young women, in particular, complain of being subjected to constant scrutiny by others, a perception amplified by the affordances of the smartphones as well as the platforms themselves; the feeling of being judged all the time, which turns into a kind of perpetual micro-surveillance device, is “[...] not simply done by others to the self, but equally is applied to one's own appearance, requiring a distinctive critical gaze on the self” (Gill, 2021, p. 1390). In fact, this mechanism engages the girls in a continuous work on themselves, their consumption and their bodies, in order to align the image shared through social media with others' expectations, whether it is the peer group to which they belong, or the followers spread across the different platforms. However, as we will see in more detail in the following paragraphs, this striving for perfection goes hand in hand with a compelling demand for authenticity, forcing women to come to terms with what Duffy and Hund (2019) define as a *gendered double bind*, namely not having to be neither too real nor not real enough.

In such a media scenario, the production of new female subjectivities is also faced with the increasing visibility gained by social media influencers, online celebrities able to garner a large number of followers on several digital platforms, from personal blogs to Instagram, Tik Tok or YouTube. As Duffy et al. (2022) point out, the gendered narratives and discourses circulating in these communities construct or reproduce ideals and patterns of femininity, sometimes regressive and traditional, with which young women have to deal, often experiencing the feeling of being wrong or inadequate.

In the light of such considerations, the paper aims to investigate the production and re-production of female subjectivity on social media, analyzing, in particular, the specificities of the Italian context in order to understand how the gendered narratives and imaginaries proposed by the country's most followed female influencers are structured between the online and offline worlds. In this perspective, the first part of the paper will focus on the elements characterizing, in today society, the media representation of female identity, while the second section will analyze the figure of female influencers, in order to highlight the characteristics of the relationships they are able to build online and offline with their female audience. Finally, the last section of the article will focus on examining the female subjectivity pattern represented by these celebrities, drawing on an analysis of two of the most popular social media influencers in Italy, Chiara Ferragni and Benedetta Rossi. By reflecting on these far from overlapping figures, we will try to understand how their personal/professional paths can be an example of the emergence of that kind of postmodern (and neoliberal) femininity at the center of gender studies reflection.

## Past and emerging gender stereotypes in the media

For some years now, mainstream media construction of feminism (and femininity) has started to emphasize its stylish, glamorous and hip character, rather than highlight its capability to shed light on the complex power relations characterizing contemporary society; from the 1990s onwards, as Gill (2016) remarks, even if gender issues increasingly take up space within the mediated public sphere and popular culture, they are addressed in a simplistic and often trivial way, turning them into one of many topics to talk about in order to seem up-to-date and cool.

In the field of gender studies, several analyses have shown how the reflection on femininity has become intertwined with the multiple political, social and cultural readings of postmodernity, ending up reflecting some of its value and, in some cases, ideological orientations. Gill (2007b, 2016) and McRobbie (2004) have addressed, for instance, the thorny implications of the postfeminist model of femininity, underlining how in newspapers, magazines, on television or on web the tropes of freedom, choice and achieved equality replaced the traditional repertoire of feminist politics and language^4^. What is widely criticized about the postfeminist approach is its focus on women as individualized subjects and therefore disembedded (Giddens, 1991) from communities and social groups, thus depriving public feminist thinking of its effectiveness as a critique of inequalities and traditional patriarchal power structures. In fact, it is precisely the spread of a *postfeminist sensibility* (Gill, 2007b) that has influenced the social image of women as conveyed by the media, emphasizing the drive for self-realization, independence and capacity to act; it is that *choice feminism*, as Hirshman (2006) has defined it, which underlines the freedom of individuals to construct their own biographical path in full autonomy, reducing the role of any type of structural or cultural conditioning with respect to the choices available to them. It is essentially a perspective that attributes to women the capability to negotiate choices irrespective of the patriarchal structures and gender inequalities that had so far governed their lives (Budgeon, 2015).

Likewise, Rottenberg (2014, p. 2018) adopts the expression *neoliberal feminism* to describe a counterpart cultural approach that gives women full responsibility for managing their own lives (and bodies), eliminating from feminist discourse any reference to collectivity, social justice and inequality: “I began to call this form of feminism neoliberal feminism, since [...] it disavows the socio-economic and cultural structures shaping our lives. This feminism also helps to spawn a new feminist subject, one who accepts full responsibility for her own well-being and self-care” (Banet-Weiser et al., 2020, p. 7). In fact, this kind of feminism driven by the rules of the market, which, not surprisingly, has found wide space in the media landscape, focuses on the individual empowerment mechanisms, pushing women to focus on themselves in order to achieve a longed-for balance between personal/affective life and professional achievement^5^. It is precisely such a model of “having it all” (Campo, 2005) that indicates for young women the goal of taking on the role of career woman without having to sacrifice either their femininity or their desire to have children, combining, in an ideal yet unrealistic union, the image of the impeccable mother/homemaker and that of the successful worker. According to this approach, through hard work and perfect organization of their days, women can aspire to what Campo (2005, p. 64) calls “the ultimate trifecta”, namely career (and their right to economic and social independence), marriage and children, while retaining their femininity^6^. However, the widespread diffusion of this gender imaginary, especially through media, advertising and the internet, risks perpetuating unrealistic feminized norms and stereotypes, offering an idealized vision of womanhood that symbolically aims to bring together (post)feminism and femininity.

In the internet context, social media also contribute to fueling similar gender expectations by conveying women's ideal images that are often far away from reality. As will become clear later on, female influencers themselves tend, in many cases, to give a misleading representation of female subjectivity by resorting to dissimulative means to project an image of accomplished and successful women; as Duffy et al. (2022) highlight, the dream of the ultimate trifecta is alive and well on Instagram. If, on the one hand, the increasing prominence of women in the cultural landscape of social media has popularized many feminist claims (often turning them into successful hashtags)^7^, on the other hand, it has partially emptied them of their political meaning, blurring the will to oppose gendered power structures and the patriarchal structure of society. This *popular feminism* (Banet-Weiser, 2018) that is all the rage in the media^8^, on the one hand fits in perfectly with the neoliberal values of self-enhancement, ambition and female entrepreneurship, while on the other hand it shares with the *postfeminist sensibility* the focus on the issue of individual choice and responsibility. In fact, gender issues also seem to be affected by the broader social process that pushes individuals to deal with systemic contradictions in an individual way (Beck, 1992): the incentive to work on oneself as a solution to gender inequalities transforms a structural problem of equal opportunities and social justice into a personal issue that every woman has to take on.

By conveying a vision of female subjectivity that is assertive but devoid of the most divisive elements, especially in relation to gendered power relations embedded in social structures, mainstream media and digital platforms contribute to the production of a gender imaginary that is in some ways depoliticized, bringing to the forefront the image of a confident and flexible woman, who also chooses the consumption realm as the setting for the exercise of her agency. Indeed, a neoliberal version of the notion of Girl Power has found its place in the media system, i.e. a depiction of femininity that enhances her affirmative and self-empowering aspects, while at the same time softens her drive to change gendered power relations within society. Rather than recognizing women as sociopolitical actors, the depoliticized versions of Girl Power offered by the dominant media institutions produce barriers to girls' active citizenship and political engagement, as they are told that power and gender equality have already been achieved; “by presenting a world with no need for social change, this use of the Girls Power discourse fails to provide girls with tools to understand and challenge situations where they experience sexism and other forms of oppression” (Taft, 2004, p. 73)^9^. It is clear how such an approach produces a type of female subjectivity that is tough and ambitious, but not conflictual, reinforcing the stereotype that sees the “happy feminist” at the very center of *popular feminism*, a figure of a woman diametrically opposed to that of the “feminist killjoy” (Ahmed, 2010) who, in public perception, had animated the second wave of feminism in the 1970s.

From our point of view, however, it would be wrong ascribing the female presence on social media to a unitary model, since digital platforms host a plurality of representations and gender models; the universe of influencers itself is definitely differentiated^10^, allowing users to experience through them multiple modes of recognition and belonging. As we will see in the following paragraphs, in fact, underlying the dynamics that govern the media presence of influencers and determine their success with audiences, online as well as offline, can be found both identification and similarity processes, as well as aspirational motivations.

## Social media influencers and building an intimate relationship with the female audience

The central role of social media in the communication ecosystem has grown hand in hand with the ability of users to publish original and creative content, using tools that allow them to narrate their daily lives or opinions in the first person. It is in this context that the figure of the social media influencer has established itself, as a character capable of converting the notoriety originally acquired on digital platforms into the offline dimension. As Abidin (2016, p. 3) points out, these are internet users “who accumulate a relatively large following on blogs and social media through the textual and visual narration of their lives and lifestyles, engage with their following in digital and physical spaces and monetize their following”. At an early stage, their communicative model aims at minimizing the cognitive and emotional distance with other users, thus choosing a deliberately simple and informal expressive style (Khamis et al., 2017); once they achieve notoriety, the communicative relationship changes and the distance with followers tends to widen. If, in the first phase, the popularity of influencers is linked to their ability to involve users through the account of their own personal experiences, blurring the boundary between public and private sphere, thus fostering the triggering of an identification mechanism (Marwick, 2013), subsequently some of them take on the features of true mainstream celebrities, capable of occupying the center of media scene and transforming the notoriety gained by dint of likes into a profession (for example, by associating their image with brands and products to be sponsored). As we will see in more detail in the next paragraph, this is the case of Chiara Ferragni, one of the world best-known fashion influencers, who started her career during university by posting daily pictures of her outfits on a blog and has now garnered almost 28 million followers on Instagram admiring her celebrity lifestyle^11^.

The influencers' ability to build an intimate relationship with their followers (Abidin, 2015), e.g. by publishing content in which people can easily recognize themselves or can trigger aspirational dynamics, is crucial. Indeed, at the basis of the successful performance of these figures is their ability to present themselves as reliable partners for an interested and participating audience, with whom they can establish a bond based on trust and on the ostentatious authenticity of their behavior^12^; also in order to assert themselves as effective advertising testimonials, influencers must appear commercially reliable, yet transparent, adopting consumption choices consistent with their own image and lifestyle (Mortara and Roberti, 2018).

Focusing the analysis on the female audience and its relationship with female influencers, it emerges how it is precisely a feeling of familiarity and closeness that characterizes the emotional connection between followers and influencers, who also use the account of their everyday life to strengthen this bond. In this context, sharing personal information creates a special relationship of trust, since celebrities are perceived as real and honest. While it is true that sharing information and personal stories is used to build a sense of intimacy with the audiences (Marwick and Boyd, 2011), it is equally clear that the *perceived intimacy* does not mean accessibility or emulation^13^, since many of these glamorous personalities lead lives that are objectively very different from those of most internet users.

What we would like to highlight here, however, is how these female figures also contribute to the construction of a common female imaginary, offering the audience that follows them a personal representation of what it means to be a woman in today society. In a continuous swing between the online and offline dimensions, the influencers are engaged in a relentless identity work, using the contents published on digital platforms to design and thus project their self-confident and emancipated image of women also in the offline dimension. However, as much as the successful femininity models available in the media are rather heterogeneous, scholars such as Favaro and Gill (2018) and Banet-Weiser (2018) stigmatize the kind of feminist sensibility circulating in the mediascape (and in social media) by labeling it as *celebrity feminism* or even *glossy feminism*^14^ and see the media system as “[....] a key cultural site for the re/production of normative, limited and limiting gender and sexual identities and relations” (Favaro and Gill, 2018, p. 40). These critics see *celebrity feminism* as a kind of cultural tendency with no real political commitment, a fad that prompts many well-known women, Hollywood stars and popular celebrities to take a stand on certain gender issues or to explicitly declare themselves as feminists^15^. According to Farris and Rottenberg (2017), if feminism has finally become legitimate in the popular imagination, at the same time has turned into an *unexpected source of social capital*, a fashionable trend, we might say, rather than a real political and ideological positioning. From this perspective, references to vague feminist values appear more as a strategic tool used to enhance one's public image than as an expression of real commitment.

Indeed, the Instagram profiles of the world's most popular female influencers^16^ reproduce a female subjectivity characterized by confidence, assertiveness and focus on self-care, but lacking the willingness to question hegemonic power structures that has traditionally symbolized female activism since the 1970s. Images of women striving to achieve their professional goals without giving up a rewarding personal and emotional life are prevailing on social media: these are women who speak to us about confidence, empowerment and self-esteem without ever dwelling on the structural roots still underlying gender inequalities. According to Gill and Orgad (2017), we are facing a psychological reading of femininity, a cultural approach that makes each woman responsible individually, spurring her to strive for greater self-confidence; by proposing individual solutions to structural problems, patriarchal society hides with the psychological language of empowerment, choice and self-responsibility the constraints of a social structure still lacking in gender equality. As the scholars write: “the confidence culture exculpates social, economic and political forces for their role in producing and maintaining inequality and instead places the emphasis upon women self-regulating and finding the ‘solutions' to their problems within a newly upgraded form of confident subjectivity. Consequently, it turns on its head the notion that the personal is political, and turns away from political critique [...]. Despite its apparently warm and affirmative address to women to believe in themselves, ‘lean in', [...] it works by locating the blame and responsibility for all difficulties and challenges in women themselves” (Gill and Orgad, 2017, p. 29).

A second feature that stands out from the Instagram profiles of these celebrities is the almost total absence of any reference to a collective subject or collective political action: the clearest claims, which mainly revolve around the concept of self-empowerment, refer to an individual dimension and are declined as personal struggles for one' own affirmation, for one's freedom of choice. With the partial exception of the campaigns against sexual harassment and rape culture, which began to spread virally as hashtags on social media, the general impression is that this generation of influencers does not deeply perceive the existence of a collective horizon of meaning, continuing instead to pursue a simplified gender discourse, which hinges on individualism and personal agency. In this sense, the female subjectivity construction, as it is represented especially on social media, appears affected by individual rather than structural or systemic issues, ignoring, or in any case downplaying, the centrality of gender politics in the public debate. However, in this regard it is important to stress that far from being neutral platforms, social media affordances are affecting the rules and conditions of social interaction (van Dijck and Poell, 2013), leading many users to change the ways in which they represent their online identity^17^. The building of influencers' Instagram profiles is far from being unrelated to these dynamics, particularly with regard to the representation of their feminine and feminist subjectivity: as Savolainen et al. (2022) point out, many female Instagram users consciously avoid addressing feminist issues that do not fit the platform's interaction order, and even refrain from expressing political viewpoints that might trigger negative reactions in audience members^18^.

Nevertheless, these young influencers' image conveyed by social media refers to a strong subjectivity, to female figures who claim the right to exploit their own agentic capacity, albeit in an individualized dimension, setting themselves up as role models for millions of followers. For some feminist researchers, however, such instances seem to be circumscribed mainly to a specific sphere, that of consumption, as if in today society female agency can only coincide with consumer agency. In this perspective, McRobbie (2008) observes how consumer culture has progressively replaced traditional social institutions such as family and education in the process of producing and reproducing the category of girl as a certain type of subjectivity; moreover, for the scholar (McRobbie, 2008, p. 544), “[...] the sprinkling of selective, even parent-friendly, feminist values” present in consumer culture is likely to conceal the gendered power relations inscribed within those powerful consumption patterns that keep women in a subordinate position. According to this understanding, girls' social power coincides with their purchasing power, thus limiting their ability to act to the consumption domain. While we refer to Roberti (2021) for a more detailed analysis of the relationship between female agency and specific consumption experiences (referring, in particular, to the meaning young women attribute to the choice of tattooing their bodies), here we simply highlight how the freedom allowed by the wide range of consumption patterns challenges normative femininity and allows girls to go beyond gender stereotypes. Wearing a particular type of dress or listening to a specific genre of music can be a powerful communicative act, a tool for asserting one's freedom or reaffirming the kind of subjectivity in which one recognizes oneself. In their role as cultural producers, influencers freely give visibility to new female patterns, also using consumer choices to reclaim their voice and centrality in the public space. If we follow Ger and Belk (1996) in defining consumer agency as the ability to transform and play with meanings, we can then highlight the active and creative role of consumers, who are thus placed at the intersection of the production and consumption dimensions^19^. Furthermore, as Bhattacharjee et al. (2014) point out, consumer agency is a major factor in identity-expressive purchase, turning embodied consumption practices into a tool for building one's own desirable gendered identity as well. Ultimately, consumer choices are only one of the ways of self-expression and one's own freedom of choice.

## Ferragni and Co: The Italian way to female influencers

In order to complete the analysis on the female figures that fill a significant space in social platforms, and thus on the ideal of femininity they produce, it is worth briefly dwelling on the Italian mediascape. Analyzing data on the most followed Italian female influencers on social media, it emerges that the most popular are Chiara Ferragni and Benedetta Rossi^20^. The former is a digital entrepreneur who, after creating the fashion blog “The Blonde Salad” in 2009, has turned the sharing of her consumer choices and lifestyle into a profession, winning millions of followers worldwide with her almost live account of her daily life. Benedetta Rossi, on the other hand, is a food blogger who, from the beginning, built her success on recipe videos posted on YouTube and on the “Fatto in casa da Benedetta [Made at home by Benedetta]” website, quickly achieving such popularity, albeit on a national level, that she obtained her own television show.

Although representing, as we shall see, two rather different models of women, it is possible to identify in their online activity some of the elements that specifically characterize the figure of the influencer, starting precisely with the strategy used in the relationship with followers. As Baym (2015) suggests, social media facilitate that “relational labor” functional to the building and maintenance of relationships with one's own audience, thus strengthening the emotional connection between fans and influencer. As we have pointed out, social celebrities also resort to the account of mundane details about personal lives in order to engage and involve users, making even more uncertain and blurred the boundary between the private dimension of their lives and the public one. Ultimately, this is an effective communication strategy that allows influencers to establish continuity in their relationship with followers, enhancing that feeling of intimacy and emotional closeness on which identification processes are built. In the same vein, both influencers alternate interactions managed through digital platforms with moments of in-person meetings with their fans, in formal and informal settings, thus reinforcing the feeling of contiguity of the communicative experience from the online to the offline sphere: “these physical space interactions complement digital space engagements because influencers are expected to perform their personae in congruence with depictions they have displayed on their blogs and social media. As such, the intimacies fostered and negotiated in digital platforms are transferred to physical settings, in a feedback loop that amplifies the sense of intimacy followers feel toward influencers” (Abidin, 2015, p. 7).

But from our viewpoint it is even more interesting to note how the Italian social celebrities embody two very connoted and distinctive models of women, to the point of appearing almost paradigmatic figures; Chiara Ferragni depicts through the media that affirmative and self-confident femininity that aims to achieve “the ultimate trifecta” (Campo, 2005), an influential entrepreneur, aware of her own capabilities, who chooses her own objectives without renouncing her affective/family life. Exemplifying that model of subjectivity typical of *neoliberal feminism*, which encourages women to empower themselves as the only ones responsible for their own successes and failures, outside of any call to collective action (Bartoletti, 2020), on her social profiles Ferragni alternates glossy images of her professional commitments with moments of family life^21^, without giving up exhorting her followers to dedicate themselves to taking care of their own outer appearance. The influencer apparently effortlessly manages this complicated balance between the roles of testimonial for important brands, loving wife and mother, proposing, in fact, to users a rather idealized model of a woman far removed from reality, a model in which entrepreneurship as women's empowerment, individualized feminism and self-branding (Marwick, 2013) seem to coincide. In fact, as Pruchniewska (2018) points out, adopting the language of individualism, autonomy and choice means, de facto, *reshaping feminism into postfeminism*: thus, women's right to choose and commitment to gender equality, issues to which Ferragni refers on more than one occasion, are declined in a generic and normalized way, lacking in any case those political references that traditionally characterized the struggle against gendered structural inequalities^22^. But social media can also be a tool for strong protests and personal attacks that, in the case of Ferragni, focus precisely on the updated version of the ideal of “having it all” that she proposes to the outside world; in fact, many users, mostly women, are pointing the finger at these representations of femininity, which are labeled as unrealistic, misleading or unattainable for those who do not have the same economic resources as the influencer. As Duffy et al. (2022) state, for these communities of anti-fans, those “fake” projections of career/family/aesthetic perfection perpetuate unachievable expectations for women and, further, represent regressive or un-feminist narratives.

However, in spite of the rhetoric of authenticity that characterizes the social profiles of many influencers, from a commercial point of view Ferragni appears to be well aware of the market rules, aiming to accurately produce and re-produce the model of woman that her followers would like to be, a style icon who has been able to build her own character one step at a time, as one does with a successful brand^23^. Framed within this logic, the rhetoric of “having it all” of which Ferragni is an emblem, reveals itself to be part of a carefully constructed, skillfully managed and constantly renegotiated self-brand project. The family pictures routinely posted by the influencer on social media also play an important role in this marketing strategy, since they prove functional in strengthening the figure of Ferragni as an all-round woman, capable of moving on several levels. Starting from the marriage with rapper Fedez and with the birth of the couple's two children, we have witnessed the progressive strengthening of “#The Ferragnez” brand, managed as a true strategic asset capable of increasing the influencer's visibility and commercial power.

Benedetta Rossi, on the other hand, embodies a more traditional and, in some ways, reassuring female subjectivity. Although she still represents an example of an established and recognizable woman within the Italian mediascape, she has achieved great popularity on social media by emphasizing the image of the spontaneous and genuine housewife, who seraphically shares recipes and cooking tips with followers. Referring to her husband Marco and their family life, described through the many pictures posted on social media, reinforce the feeling of closeness with consumers, also transforming the couple's image into a marketable product; in a number of Instagram stories, for instance, it is Marco who starts cooking in their home kitchen, while his wife Benedetta takes videos, as a typical married couple would do^24^. Indeed, Rossi uses a communication style tuned to the register of simplicity, a genuine manner that makes her a familiar presence for the millions of women who follow her on television and through digital platforms. Compared to Ferragni, capable of triggering aspirational dynamics in audiences, Rossi embodies a more achievable female model, which does not aim to question traditional gender roles or challenge the legitimacy of power relations. Indeed, one could speak of a rather conventional media representation of female subjectivity, which fits into the productive format of the so-called *cooking shows*, where women are generally seen in the role of mothers/wives engaged in cooking for the family^25^. As a result of this kind of media imaginary, the domestic setting and the presence of a female figure in a kitchen reinforce the atmosphere of intimacy established with the audiences, activating in them an effective mechanism of identification with the protagonists of the shows. However, if, a few years ago, Oren (2013, p. 25) could rightly claim that “in television, as in other corners of the popular imagination, gendered divisions around cooking remain largely over place and intention: women cook at home for their family and loved ones, men cook in public, for pay, and mostly for (adoring) strangers”, today the reality seems to have somewhat changed, as the web and social media have begun to expand the spaces (and roles) available to women. *Cooking shows* themselves, albeit slowly, have taken new paths, from factual entertainment to talent shows, exploiting precisely the opportunities and synergies allowed by digital platforms. As to Benedetta Rossi, it is possible to highlight the existence of a twofold expressive register: if it is true that the home setting and the informal atmosphere that characterize her videos refer to a communicative choice aimed at reducing the emotional distance from the users and favoring their identification, it is equally clear that the influencer represents a winning entrepreneurial model, as she was able to make the most of the social media's capabilities. As such, Rossi provides an example of a woman who has been able to use her professional skills to build a female success story. Ultimately, it seems to emerge that, while playing on two different fronts, both Ferragni and Rossi have been able to offer their followers a pattern of femininity to which they can refer, actively contributing to the production of those *multiple female imaginaries* that very different women today can draw inspiration from.

## Discussion and conclusion

As we already discussed, media play a fundamental role in constructing and making visible the different patterns of femininity; digital platforms, in particular, have multiplied the spaces available to women, making it easier for them to access the “places” of cultural production. As also the description of the goals set by the United Nations for favorable Sustainable Development underlines, information and communication technologies can prove to be an extraordinary tool for promoting gender equality and women's empowerment^26^.

In such an intertwined context, social media influencers occupy an even more relevant position, as they collaborate in the production of texts and discourses that shape the perception of gender identities in the audience. With their public visibility, they also perform a function of *legitimizing cultural practices and symbols*, allowing internet users to express their gender subjectivity with relative freedom. For this reason, the question of the models of femininity that social media convey to the public remains highly topical, since, as we have pointed out, media depictions have a significant impact on gendered narratives and discourses. In this perspective, analyzing the role played by female social media influencers may allow us to better understand which models of action inspire women today and define their collective imaginary. As Gill (2021), among others, has pointed out, on social media most young women are experiencing the feeling of being inadequate, making the need to understand what dynamics regulate the media production of normative gendered identities even more compelling.

Our analysis has shown that the ability of influencers to construct depictions of femininity in which women can mirror themselves is linked, first and foremost, to the intimate relationship that they are able to establish with their followers; these *social celebrities*, as we have defined them, use the narrative of their everyday life to give continuity to the bond established with their audiences and to accredit themselves as credible partners in the communicative exchange. In fact, the dialogue enabled by digital media and the potential two-way nature of the interaction reinforce the feeling of intimacy and closeness in users, facilitating their willingness to mirror themselves in the ideal of woman embodied by the influencer. Taking full advantage of the process of disintermediation favored by the spread of digital technologies, influencers have built a direct communication channel with their audiences, gradually learning to manage relations with increasingly active and interconnected subjects, users who have been able to naturally adapt their actions to the new two-way interaction modes^27^.

Several scholars who refer to the field of *gender and feminist media studies* have highlighted how the female subjectivity depicted on social media is predominantly inspired by an ideal of digital entrepreneurial femininity steeped in neoliberal and postfeminist ideology (Rottenberg, 2018; Banet-Weiser et al., 2020), a social representation that values women's independence, individual capabilities and self-empowerment. The two Italian influencers examined, Chiara Ferragni and Benedetta Rossi, although very different one from the other, reflect this model, since both, through a strategic use of social media, have created a successful entrepreneurial project, turning their passion, respectively for fashion and cooking, into a profession. Similar patterns of *corporate feminism*, which have turned online interactions into a lucrative economic practice, consider one's personal life and experiences as strategic assets, to the extent that the boundaries between the public and private spheres are becoming increasingly blurred (Petersson McIntyre, 2020).

Nevertheless, the analysis of the femininity model proposed through digital platforms by Ferragni and Rossi must necessarily take into account other significant elements, starting with the prominence they have assumed in the collective imaginary. If it is true that, in some ways, the two celebrities represent somewhat stereotyped and one-dimensional figures – one committed to updating the prototype of the “having it all” woman and the other to outlining the profile of the 2.0 housewife – it is equally evident that they have contributed to legitimizing and giving greater visibility to female figures in the Italian social media landscape. These are, in fact, two women who, in different ways and styles, have succeeded in claiming their own voice in the public arena, effectively using those mechanisms of *transmedia storytelling* (Scolari, 2009) capable of guaranteeing the emotional engagement of followers. As we know, using different media and languages leads to a much deeper involvement by the audience, who feels an active part of the story created for them on the different digital platforms.

In conclusion, it is necessary to emphasize the need to enrich the reflection on the social construction of gender with new analytical tools, in order to gain a deeper understanding of the mechanisms of production of female subjectivity in different social and cultural contexts; although today studying gender representations in the media is still fundamental, it would be important, in our opinion, to examine in depth the *practices and contexts of consumption* in which women are protagonists, in order to promote the development of a more critical and conscious femininity, also in relation to issues of gender equality.

## Ethics statement

Ethical review and approval was not required for the study involving human subjects in accordance with the local legislation and institutional requirements. Written informed consent to participate in this study was not required from the subjects in accordance with the national legislation and the institutional requirements.

## Author contributions

GR conceptualized the contribution, wrote the paper, reviewed the manuscript, and provided the critical revision processes.

## Conflict of interest

The author declares that the research was conducted in the absence of any commercial or financial relationships that could be construed as a potential conflict of interest.

## Publisher's note

All claims expressed in this article are solely those of the authors and do not necessarily represent those of their affiliated organizations, or those of the publisher, the editors and the reviewers. Any product that may be evaluated in this article, or claim that may be made by its manufacturer, is not guaranteed or endorsed by the publisher.

## Footnotes

^1^As Davis and Jurgenson (2014, p. 476) also write: “the collapsing of social contexts together has emerged as an important topic with the rise of social media that so often blurs the public and private, professional and personal, and the many different selves and situations in which individuals find themselves”.

^2^See, just as an example, the work of McRobbie (2004, 2008), Gill (2007a, 2012) and van Zoonen (1994, 2010).

^3^Gill (2021, p. 1388) states: “there were also a huge array of dilemmas that related to other issues - not least how to be, how to live and how to present oneself in contemporary mediated culture, in which they felt both under ubiquitous surveillance and subject to constant judgment - but from which they could not readily escape without also losing the chance to feel part of the pleasurable flow of life, knowing what is happening, seeing and being seen”.

^4^In reflecting on postfeminism, McRobbie (2004), in particular, draws attention to a “double entanglement” that “comprises the co-existence of neo-conservative values in relation to gender, sexuality and family life […] with processes of liberalization in regard to choice and diversity in domestic, sexual and kinship relations”, turning it into a form of Gramscian common sense.

^5^It is with regard to such a cultural shift in feminist politics that Banet-Weiser et al. (2020, p. 14) write: “[…] the issues of work-life balance are nothing new, but what is new, at least in the US, is the positing of a happy work-family balance as a feminist ideal and as the signifier for emancipation for progressive womanhood”, providing a kind of heteronormative ideal that is difficult to evade.

^6^With the expression “ultimate trifecta”, Campo (2005) is referring to precisely that kind of female cliché whereby women strive to combine the care of their femininity and beauty with professional ambition and the desire to have a husband and children.

^7^We refer, just to give a few examples, to hashtags gone viral such as #metoo, #yesallwomen, #heforshe or, in Italy, #nonunadimeno.

^8^The popular feminism “appears on broadcast media, in television and advertising. It appears in popular music. In the contemporary context, it appears perhaps most urgently in social media, with digital sites such as Instagram, Tumblr, Facebook, and Twitter providing platforms for its circulation” (Banet-Weiser, 2018, p. 9).

^9^As Taft itself points out (2004), the issue of the pursued gender equality has to deal with women's claims regarding the intersectionality of gender inequalities with race, class, sexuality and ability. Regarding *intersectional feminism*, see, among others, McCall (2005), Butler (2013), Munro (2013) and Zimmerman (2017).

^10^It is worth mentioning, in this regard, that, for some years now, social media has hosted a new category of influencers, the *virtual influencers*, pixel characters created for commercial purposes by sophisticated graphic studios and endowed with a specific identity; see Andersson and Sobek (2020).

^11^This is the text of the first post published on October 12, 2009 on her blog “The Blonde Salad” and entitled “Here I am”: “Here we are at the first independent blog driven by needs of communication and personalization. After years spent on Flickr and other different web communities, I felt like I had to move on and create a space of my own. The name is “The Blonde Salad” because this blog is gonna be a salad of myself. The ingredients will be those which have always characterized me: fashion, photography, travel and lifestyle. I hope that this step forward could involve you even more”. As it is evident, at the time the blogger had not yet assumed the role of celebrity and still addressed other users by putting herself on an equal footing.

^12^Wellman et al. (2020) refer, in this regard, to a kind of *ethics of authenticity*, namely the effort by influencers to be true and loyal to one's self and personal brand and loyal to one's audience, while Pooley (2010) speaks of *calculated authenticity*.

^13^As Abidin (2015) points out, one could speak of “perceived” intimacy between the subjects engaged in the communicative exchange to highlight the difference from authentic and truly experienced intimacy forms.

^14^In Italy, Capecchi (2021) referred to the spread of a *glamorous version of feminism*.

^15^In this regard, Ghigi and Rottenberg (2019, 657) write: “if just ten years ago, the vast majority of high-profile women in the West refused to identify as feminist, today many famous actresses, singers, and politicians are publicly coming out as feminists, denouncing the widespread gender discrimination and sexual exploitation in Hollywood, the entertainment business, and the political sphere […]”.

^16^See the ranking of the most followed Instagram accounts worldwide released by Statista (June 2022): https://www.statista.com/statistics/421169/most-followers-instagram/ [accessed July 27, 2022]. According to this ranking, among women influencers, the top 3 positions are occupied by Kylie Jenner, Kim Kardashian, Kloe Kandashian. We have excluded from the analysis the social profiles of celebrities who have achieved notoriety for their activities in other fields of entertainment (such as actresses, singers, or journalists).

^17^In this regard, Savolainen et al. (2022) describe real filtering practices that female users adopt on Instagram to adapt their profile to the interaction order promoted by the platform.

^18^“The strong constraint exercised by perceived audience expectations came to light as several respondents reported holding back political views they thought were not sufficiently popular. Users […] were aware of their local followers' suspicion toward feminism and left-wing politics, and found it nearly impossible to express their political views on social media” (Savolainen et al., 2022, p. 564).

^19^Not by chance, beginning from Toffler (1980) and Ritzer (2014), the sociology of consumption has dedicated considerable space to the spread of the figure of the prosumer.

^20^The data, provided by Shareablee, are for April 2021 and refer to the Italian female influencers claiming the most interactions on social media. We have excluded from the analysis the social profiles of celebrities who have achieved notoriety for their activities in other fields of entertainment (such as actresses, singers, or journalists). For further information, see https://www.sensemakers.it/news/top-15-influencer-italiani-piu-attivi-sui-social-aprile-2021 [accessed July 27, 2022].

^21^A pattern that repeats the scheme that Duffy and Hund (2015, p. 9) had already pointed out in their research on fashion bloggers: “finally, given that ‘authenticity' and ‘realness' are governing logics in the fashion blogging community, it is perhaps not surprising that bloggers moderate representations of the ‘glam life' with images that make them seem *just like us*. The latter depictions serve as a kind of aesthetic foil to images of artful perfection that circulate in women's magazines. Of course, even these seeming moments of candor – with family, friends, and pets included – do not disrupt bloggers' well-crafted social media personae”.

^22^For example, in a live stream on her husband Fedez's Twitch channel, where the couple's earnings were being compared, Ferragni first intervened by emphasizing her own economic strength in the name of a vague Girl Power and then defused any controversy by claiming a more harmless “*Ferragnez* power”, see https://www.biccy.it/fedez-e-chiara-ferragni-guadagna/ [accessed September 2, 2022].

^23^As Pedroni (2021) underlines, through her own career path Ferragni gives followers a glimpse, albeit in the abstract, of the possibility that *ordinary people* can also become successful influencers in their turn, by virtue of the web's proclaimed democratic nature.

^24^See https://www.instagram.com/stories/highlights/18132761620283200/ [accessed September 2, 2022].

^25^On the most relevant aspects of the relationship between gender and cooking show see Rousseau (2013) and Matwick and Matwick (2014, 2017).

^26^The Sustainable Development Goals are a universal call to action to end poverty, protect the planet and improve the lives of everyone, everywhere. The 17 Goals were adopted by all UN member states as part of the 2030 Agenda for Sustainable Development; see https://www.un.org/sustainabledevelopment/ [accessed August 2, 2022].

^27^In this sense, reflecting on the concept of digital fashion, Calefato (2021) notes how the presence of influencers in the *fashionscape* and the direct intervention of consumers through the web have complemented or completely replaced traditional forms of offline communication and consumption of fashion signs, bypassing the usual mediating agencies, such as fashion magazines or traditional fashion events.
